# The cardiovascular and hypothalamus-pituitary-adrenal axis response to stress is controlled by glucocorticoid receptor sequence variants and promoter methylation

**DOI:** 10.1186/s13148-016-0180-y

**Published:** 2016-01-28

**Authors:** Ting Li-Tempel, Mauro F. Larra, Estelle Sandt, Sophie B. Mériaux, Andrea B. Schote, Hartmut Schächinger, Claude P. Muller, Jonathan D. Turner

**Affiliations:** Department of Neurobehavioral Genetics, Research Institute of Psychobiology, University of Trier, 54290 Trier, Germany; Department of Clinical Physiology, Research Institute of Psychobiology, University of Trier, 54290 Trier, Germany; Department of Infection and Immunity, Luxembourg Institute of Health, 29 rue Henri Koch, Esch-sur-Alzette, 4354 Grand-Duchy of Luxembourg; Department of Immunology, Research Institute of Psychobiology, University of Trier, 54290 Trier, Germany

**Keywords:** Glucocorticoid receptor, Alternative promoter, Single nucleotide polymorphism, Methylation

## Abstract

**Background:**

Gender, genetic makeup, and prior experience interact to determine physiological responses to an external perceived stressor. Here, we investigated the contribution of both genetic variants and promoter methylation of the *NR3C1* (glucocorticoid receptor) gene to the cardiovascular and hypothalamus-pituitary-adrenal (HPA) axis response to the socially evaluated cold pressor test (seCPT).

**Results:**

Two hundred thirty-two healthy participants were recruited and underwent the experiment. They were randomly assigned to either the seCPT group (cold water) or a control group (warm water). The seCPT group had a clear stress reaction; salivary cortisol levels and peak systolic and diastolic blood pressure all increased significantly compared to the control group. *GR* genotype (*TthIII*I, NR3C1-I, 1H, E22E, R23K, *Bcl*I and 9beta) and methylation data were obtained from 218 participants. Haplotypes were built from the *GR* genotypes, and haplotype 2 (minor allele of *Bcl*I) carriers had a higher cortisol response to the seCPT in comparison to non-carriers (20.77 ± 13.22; 14.99 ± 8.42; *p* = 0.034), as well as independently of the experimental manipulation, higher baseline heart rate (72.44 ± 10.99; 68.74 ± 9.79; *p* = 0.022) and blood pressure (115.81 ± 10.47; 111.61 ± 10.74; *p* = 0.048). Average methylation levels throughout promoter 1F and 1H were low (2.76 and 1.69 %, respectively), but there was a strong correlation between individual CpGs and the distance separating them (Pearson’s correlation *r* = 0.725, *p* = 3.03 × 10^−26^). Higher promoter-wide methylation levels were associated with decreased baseline blood pressure, and when incorporated into a linear mixed effect model significantly predicted lower systolic and diastolic blood pressure evolution over time in response to the experimental manipulation. The underlying genotype significantly predicted methylation levels; particularly, the homozygous *Bcl*I minor allele was associated with higher methylation in promoter 1H (*p* = 0.042).

**Conclusions:**

This is one of the first studies linking epigenetic modifications of the *GR* promoter, receptor genotype and physiological measures of the stress response. At baseline, there were clear genetic and epigenetic effects on blood pressure. The seCPT induced a strong cardiovascular and HPA axis response, and both systems were affected by the functional genetic variants, although methylation also predicted blood pressure reactivity. The return to baseline was predominantly influenced by the genomic sequence. Overall, the physiological response to the seCPT is controlled by an exquisite mix of genetic and epigenetic factors.

**Electronic supplementary material:**

The online version of this article (doi:10.1186/s13148-016-0180-y) contains supplementary material, which is available to authorized users.

## Background

External challenges trigger hypothalamus-pituitary-adrenal (HPA) axis activation and cortisol secretion, maintaining homeostasis and permitting adaptation [1]. Glucocorticoid receptor (GR, gene: *NR3C1*, OMIM +138040) protein isoforms and levels throughout all HPA axis tissues control glucocorticoid (GC) feedback, setting individual levels of stress reactivity and responsivity. A complex interplay of genetic and epigenetic mechanisms control GR levels, protein isoforms, and potentially the end phenotype. Twin research has suggested that part of the inter-individual differences in the stress response may be explained by genetic factors [2], and both rodent models and human studies show an environmental influence via epigenetic mechanisms [3–5]. Both epigenetic and genetic factors influence the transcriptional control of the *GR* through the series of tissue-specific promoters found upstream of the 11 alternative *GR* first exons [6–8].

It is well established that individual genetic variants of the glucocorticoid receptor affect both the basic cellular phenotypes i.e. *GR* expression levels [9] and the overall HPA axis stress response (reviewed in [10]) through either an altered GC response or sensitivity. Numerous *GR* SNPs are in a high linkage disequilibrium resulting in commonly accepted haplotypes [10] (Additional file 1: Table S1). Three haplotypes, *Bcl*I alone, *TthIII*I + *Bcl*I and N363S alone are all associated with an increased sensitivity to GCs [11, 12]. The N363S polymorphism was associated with increased BMI, raised cholesterol levels and an increased risk for coronary artery disease [12]. Inversely, two haplotypes *TthIII*I + 9β and *TthIII*I + 9β + ER22/23EK have been associated with GC resistance [13]. Importantly, there are a total of 12 known genetic variants throughout the 8 confirmed promoter regions controlling the expression of the 11 alternative first exons in the variable 5′ untranslated region (UTR) of the *GR* [9, 14]. This 5′UTR is responsible for controlling tissue-specific alternative first exon expression, overall GR levels and isoforms [6, 7, 15, 16].

Epigenetic modifications such as DNA methylation, post translational chromatin remodelling and small RNA-based mechanisms have more recently been shown to contribute both independently or together with genetic variation to gene regulation. DNA methylation is unique, as it is the only epigenetic mechanism that may regulate gene expression, is clearly propagated through mitosis, whilst retaining its function [17]. Although the associations of *GR* promoter methylation with diseases such as posttraumatic stress disorder [18] and depression [5, 19–21] are well studied, there is very little evidence on how it influences HPA axis (re)activity or any other aspect of the stress response such as cardiovascular reactivity. The available studies provide inconclusive data. High methylation levels were associated with an increased salivary cortisol response in infants [19] and with a female-specific increased cortisol secretion after stress [22]. Conversely, increased methylation levels were also associated with a decreased response to pharmacological HPA axis stimulation [23] or could be explained by differences in both education and lifestyle [24]. The effect of DNA methylation on stress-related cardiovascular reactivity remains unexplored. These studies were limited to the proximal *GR* promoter regions thought to control tissue and stimuli specific GR levels [6, 16]. The mechanisms underlying the effects of DNA methylation on gene expression are not, however, particularly well understood [25]. The longstanding association of DNA methylation with gene silencing (reviewed in [26]) does not reflect its functional complexity, orchestrating tissue-specific regulatory elements and expression patterns [27], marking alternative intra-genic promoters [28], controlling alternative splicing [25, 29, 30] and even promoting gene transcription [27, 31, 32]. The evidence currently available suggests that methylation in these regions of the *GR* do not only control the relative promoter activity, and levels of individual first exon transcripts, but also the final protein isoform and its cellular localisation [7, 16].

Genetic and epigenetic factors work together to produce the overall response, reflected in the cortisol secretion and cardiovascular system activation to an external stressor; however, neither factor acts unilaterally. Whilst epigenetic factors, particularly DNA methylation, integrate the environment experienced with the genotype [33], the underlying DNA sequence also has a large influence on methylation levels. In both genome-wide family-based genetic studies and HapMap cell lines, genetic variants affected DNA methylation without necessarily introducing new CpG methylation sites [34, 35]. However, the only *GR* data available to our knowledge suggests that there is no association between the *Bcl*I variant and methylation of the GR promoter 1F in the human placenta, although none of the other promoters or exons were investigated. However, there was a potential association between methylation, genotype and infant neurobehavioural outcomes [36].

In this study, we investigated the relative contribution of genetic variants and promoter methylation of the *GR* to both cardiovascular and HPA axis stress reactivity. In a cohort of healthy adults, stress was induced using the socially evaluated cold pressor test (seCPT), and cardiovascular reactivity was assessed from heart rate (HR) and systolic and diastolic blood pressure (SBP/DBP) changes. HPA axis reactivity was assessed from salivary cortisol. We identified associations between promoter methylation and genetic variants and analysed how these impact cardiovascular as well as HPA axis activity in response to the seCPT.

## Results

### Study population and randomisation

**Fig. 1:**
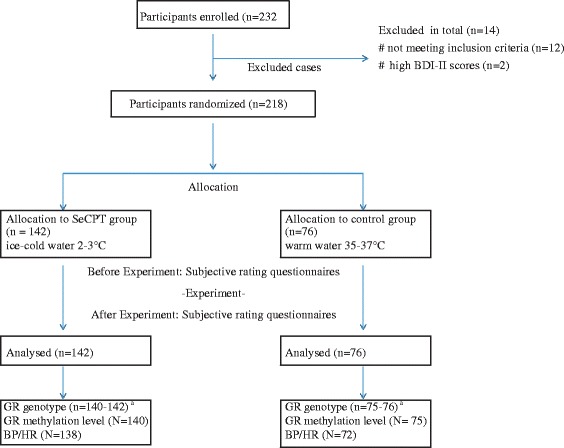
Recruitment summary for all donors contacted, participating, exiting and analysed after completion of the study

A homogenous cohort of 232 undergraduate students with minimal lifestyle differences were recruited, and data from 218 (103 males and 115 females) were analysed (Fig. 1). Two participants had BDI-II scores of 25 and 26, indicative of moderate depression and were excluded from all subsequent analyses. The mean BDI-II score of 5.976 was within the minimal range (0–13). Although the mean BDI-II score for female participants was slightly higher (*p* = 0.011) compared to males, no difference has been found between the seCPT and control group (*p* = 0.438). When included in the study, participants were given a date for the experimental session. Upon arrival on that date, they were assigned to either the seCPT or control group in a 2:1 alternating order. At baseline, there were no group differences in any of the variables tested (Table 1).

**Table 1:** Summary of participant characteristics and group gender repartition

	seCPT group	Control group	Full population	*p* value^a^
Sex (male)	70	35	105	0.736
Age	22.89 ± 2.7	23.27 ± 3.0	23.01 ± 2.8	0.356
BMI	22.1 ± 2.3	22.5 ± 2.4	22.2 ± 2.3	0.243
BDI	6.1 ± 5.3	5.5 ± 4.5	6.0 ± 5.1	0.438
Arousal (0–100)	20.1 ± 19.3	23.2 ± 20.1	23.8 ± 19.5	0.769
Stress (0–100)	25.2 ± 21.8	22.4 ± 21.3	24.3 ± 21.6	0.379
Anxiety (0–100)	12.1 ± 15.6	7.6 ± 9.5	10.6 ± 14.1	0.010
Tension (0–100)	17.8 ± 4.8	17.6 ± 5.2	17.7 ± 4.9	0.803
Activity (0–100)	26.3 ± 6.5	27.6 ± 6.1	26.7 ± 6.4	0.153
	Males	Females	Analysed population	
Complete cohort	103 (47.2 %)	115 (52.8 %)	218 (100 %)	
seCPT	70 (66.66 %)	77 (68.14 %)	147 (66.97 %)	
Control	35 (33.33 %)	36 (31.86 %)	72 (33.03 %)	

^a^Comparison of seCPT vs control Gp

### seCPT induced a physiological and subjective psychological stress response

As previously reported [37], salivary cortisol levels were significantly increased in the seCPT group compared to the control group (*p*_AUCg_ = 0.001; *p*_AUCi_ < 0.001, *t* test). Peak SBP and DBP levels were significantly increased from baseline in the seCPT group compared to the control group. However, both SBP and DBP were comparable between the seCPT and control groups in both the baseline and recovery periods (*p* = 0.299 and *p* = 0.712, ANOVA between the seCPT and the control group). Bivariate analysis by sex showed a significantly greater increase in SBP levels in men (*p* = 7.02 × 10^−8^) and a trend towards a greater increase from baseline to peak levels (*p* = 0.093). There was no effect of sex on diastolic blood pressure or heart rate increase, decrease or peak levels (*p* > 0.1). Participants rated the seCPT significantly more stressful and had significantly higher levels of arousal, anxiety, activity and tension (all *p* < 0.01 paired *t* tests) compared to the control group. Subjective ratings were not dependent on gender (*p* > 0.05), although female participants tended towards increased anxiety in the seCPT group compared to control (*p* = 0.057).

### Genotype and haplotype analysis in the cohort

**Fig. 2:**
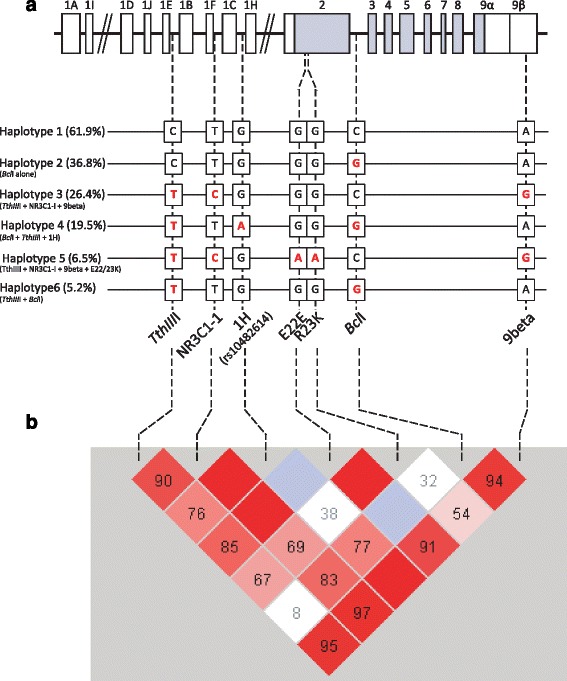
The genomic organisation, sequence variants and haplotype structure of the glucocorticoid receptor gene (*NR3C1*). **a** A schematic representation of the *NR3C1* genomic organisation. *Rectangles* represent transcribed exons. Exons 1A–1I are alternatively spliced to a common acceptor site at the start of exon 2. *White* exons are non-coding, *grey* exons represent the coding sequence. The *lower section* of the panel shows the six haplotypes observed, their constituent variants and frequencies. Minor alleles are represented by bold red letters. **b** The linkage disequilibrium (LD) structure of the *NR3C1*. LD between two variants are given by colour, *blue/grey* no LD; *white*, limited LD; *light red* to *dark red*, medium to strong LD. Numbers within the LD *diamonds* represent the value of D prime (D’) between the two loci. D’ is statistic normalised parameters of disequilibrium

*GR* genotyping was completed for *TthIII*I, NR3C1-I, 1H, E22E, R23K, *Bcl*I and 9beta in 217, 215, 218, 218, 216, 218 and 218 participants, respectively. Due to their low frequencies, both hetero- and homozygous carriers were combined into one group for R23K (GA = 18, AA = 3) and promoter 1H (GA = 55, AA = 3). Minor allele frequencies (Table 2), and LD scores (D’) (Fig. 2) were in line with previously reported data [38]. The haplotype structure was successfully created with PHASE for all the available data points, and those with frequencies above 5 % are illustrated in Fig. 2. Haplotype 1 (C-T-G-G-G-C-A) had a frequency of 61.9 % and consisted of the major alleles of each SNP. Haplotype 2 (C-T-G-G-G-G-A) with 36.8 % contained the minor alleles of *Bcl*1. Haplotype 3 (T-C-G-G-G-C-G) included the minor allele of T*thIII*1, NR3C1-I and 9beta and showed a frequency of 26.4 %. Haplotype 4 (T-T-A-G-G-G-A) with 19.5 % contained the minor allele of T*thIII*1, 1H and *Bcl*1. Haplotype 5 (T-C-G-A-A-C-G) with 6.5 % contained the minor allele of T*thIII*1, NR3C1-I, *ER22E*, *R23K* and 9beta. Haplotype 6 (T-T-G-G-G-G-A) included the minor allele of T*thIII*1 and *Bcl*1 showed a frequency of 5.2 %. The haplotype structure was similar to that previously reported [9, 38].

**Table 2:** Descriptive data of *NR3C1* single markers in 218 participants using Haploview

Marker	Position	obsHET	predHET	HWpval	% geno	MAF	Alleles
*TthIII*1	−142766894	0.463	0.458	1.0	99.6	0.355	C:T
NR3C1-I	−142763714	0.356	0.331	0.368	98.7	0.209	T:C
1H	−142762357	0.241	0.232	0.8032	100.0	0.134	G:A
E22E	−142760532	0.075	0.072	1.0	100.0	0.037	G:A
R23K	−142760530	0.084	0.105	0.0437	99.1	0.055	G:A
*Bcl*1	−142758768	0.408	0.462	0.0988	100.0	0.362	C:G
9beta	−142637814	0.307	0.306	1.0	100.0	0.189	A:G

*HWpval p* value for Hardy-Weinberg equilibrium, *MAF* minor allele frequency, *obsHET* observed heterozygosity, *position* chromosomal location, *predHET* predicted heterozygosity, *% Geno* genotyping frequency

### Haplotype associations with HPA axis reactivity

AUCg and AUCi were used as the dependent variable in separate between-participants ANOVAs with the factors seCPT group and genotype (with each SNP and each haplotype as the genotype factor). AUCg was influenced by a significant interaction of haplotype 2 (*Bcl*I alone) and seCPT group (20.77 ± 13.22; 14.99 ± 8.42; *p* = 0.034). Scrutinising the structure of this interaction effect, effects analyses showed that carriers of haplotype 2 had a significantly higher AUCg than non-carriers in the seCPT group (20.77 ± 13.22; 14.99 ± 8.42; *p* = 0.003), whereas carriers and non-carriers did not differ significantly in the control group (Additional file 1: Table S3).

### Association of haplotypes with HR and SBP

To evaluate the influences of genotypes and haplotypes on SBP and HR, a series of between-participants ANOVAs were performed. The results are summarised in Additional file 1: Table S3. There were significant main effects on the baseline, peak and recovery with higher HR for carriers of haplotype 2 (72.44 ± 10.99; *p* = 0.022, 74.05 ± 12.25; *p* = 0.023, 69.50 ± 9.48; *p* = 0.027, respectively) as compared to non-carriers (68.74 ± 9.79; 70.65 ± 11.74; 66.20 ± 9.28). We observed, independent of the experimental group, a significantly higher decrease of HR after the water task in carriers of haplotype 3 compared to non-carriers (6.93 ± 9.80; 3.55 ± 7.03; *p* = 0.016).

**Fig. 3:**
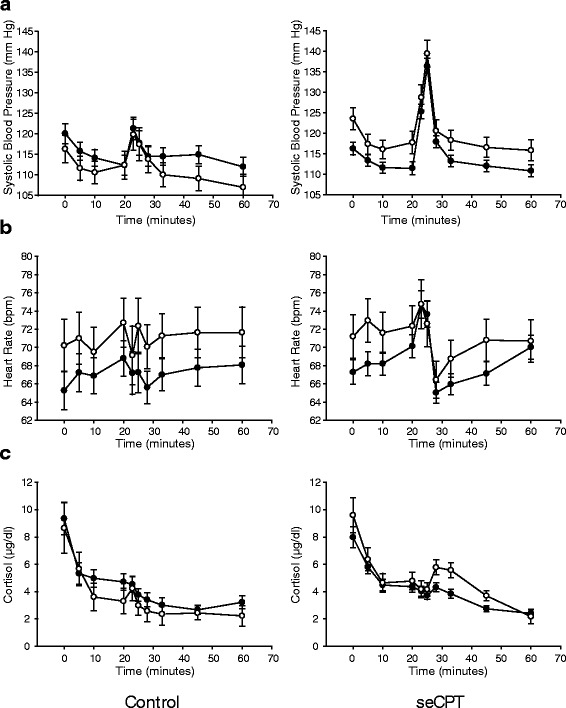
Haplotype 2 (*Bcl*I alone) effects of the warm (control) or ice-cold water (seCPT) condition on SBP, heart rate and cortisol over the course of the experiment. **a** Systolic blood pressure in millimeter of mercury after the control (*left panel*) or cold water (*right panel*). **b** Heart rate in beats per minute after the control (*left panel*) or cold water (*right panel*). **c** Salivary cortisol levels in the control (*left panel*) or cold water (*right panel*). The seCPT or warm water was administered at 23 min and lasted 3 min. In all panels, *filled circles* are homozygous wild-type (CC) participants and *open circles* are homozygous minor allele (GG) participants. Data are the mean ± the standard error of the mean

Homozygote carriers of the minor G allele of the SNP *Bcl*I showed higher baseline HR (72.51 ± 11.57; 68.39 ± 9.13; *p* = 0.048). “Bpm increase” was influenced by a significant interaction of *Bcl*I and seCPT group (−0.76 ± 7.89; 4.61 ± 9.02; *p* = 0.029). Simple effects analyses showed that homozygous carriers of the C allele had a significantly higher increase than homozygous carriers of the G allele in the seCPT group (−0.76 ± 7.89; 4.61 ± 9.02; *p* = 0.006), whereas CC and GG carriers did not differ significantly in the control group (Additional file 1: Table S3). In our study, the G allele of *Bcl*I is a risk allele for higher HR. Carriers did not respond to a stressor in the same way as the carriers of the C allele, that is, with an increase in HR (Fig. 3).

There was an interaction between haplotype 2 and group regarding SBP baseline (115.81 ± 10.47; 111.61 ± 10.74; *p* = 0.048). Only in the seCPT group, carriers of haplotype 2 had a significantly higher “SBP baseline” than non-carriers (115.81 ± 10.47; 111.61 ± 10.74; *p* = 0.025). This effect of haplotype may have emerged only in the seCPT group because of the higher participant number in there. There was also an interaction between haplotype 3 and group regarding the recovery SBP (112.54 ± 11.22; 116.41 ± 10.68; *p* = 0.019). Non-carriers in the seCPT group had higher recovery SBP than the non-carriers in the control group (116.41 ± 10.67; 112.75 ± 11.88; *p* = 0.049), whereas carriers in both groups did not differ.

### GR promoter methylation levels and distribution in the cohort

**Fig. 4:**
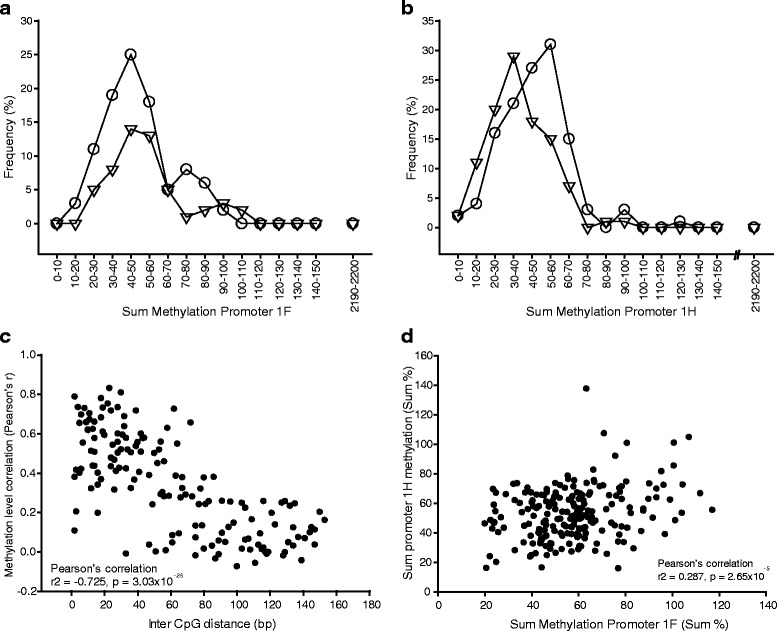
Methylation of the *NR3C1* promoters 1F and 1H. **a** Frequency distribution of the sum of the methylation throughout promoter 1F. Female donors, *open circles*; male donors, *open triangles*. **b** Frequency distribution of the sum of the methylation throughout promoter 1H. Female donors, *open circles*; male donors, *open triangles*. **c** Pearson’s correlation coefficients were calculated for all CpG pairs and subsequently plotted against the physical distance measured in nucleotides, demonstrating that the closer two CpG nucleotides are, the stronger their correlation in methylation levels. Each data point represents Pearson’s correlation coefficient for one pair of CpGs from all donors. **d** Pearson’s correlation in methylation levels between sum methylation levels in promoter 1F and 1H. Each data point represents one participant

Methylation analysis was performed on 218 participants. In total, 14 participants (6 %) were excluded, 3 due to missing methylation data, 10 for no HR/SBP/DBP data and 1 for which both data were missing. Average methylation levels of individual CpGs in promoters 1F and 1H were 2.76 and 1.69 %, respectively, and were directly comparable to previous reports from human white blood cells. Methylation levels did not exceed 14 % for any donor at any position throughout promoters 1F and 1H. As reported for previous cohorts [15], methylation levels of individual CpGs in close proximity strongly correlated in both promoter 1F and 1H (Pearson’s correlation *r* = 0.725, *p* = 3.03 × 10^−26^; Fig. 4), confirming CpG methylation levels were co-regulated over short distances, probably in small clusters.

As methylation levels correlated in clusters, promoter-wide sum methylation levels were investigated. Promoter 1H sum methylation levels were significantly higher in women than men (Mann-Whitney rank sum test, *p* < 0.01; Fig. 4), although there was no difference for promoter 1F sum methylation levels (Mann-Whitney rank sum test, *p* = 0.91; Fig. 4). This difference was maintained for methylation summed throughout the two promoters (*p* = 0.038, Mann-Whitney rank sum test). Although sum methylation levels for promoters 1F and 1H were not normally distributed (*p* < 0.001 Shapiro and Kolmogorov-Smirnov tests), there was a weak but significant Pearson’s correlation between the two promoters (*r* = 0.287, *p* = 0.65 × 10^−4^; Fig. 4), suggesting the clusters may also cover complete promoters.

For our linear mixed effects model, methylation levels, despite the potential loss of statistical power, were treated as a binary variable after a median split. After median split, the difference in methylation between the sexes were reflected in the ~60 %:40 % ratio of males to females in the low methylation group and the inverse in the high methylation group. There was no bias in their randomisation into the seCPT or control group (Table 3).

**Table 3:** Gender repartition after median split on methylation level

		Males, *n* (%)	Females, *n* (%)	Full population
Low methylation		60 (56.07)	47 (43.93)	107 (49.08)
seCPT group		42	31	73
Control group		18	16	34
High methylation		44 (39.64)	67 (60.36)	111 (50.92)
seCPT group		27	31	73
Control group		17	21	38

### Methylation level predicts SBP and DBP

**Fig. 5:**
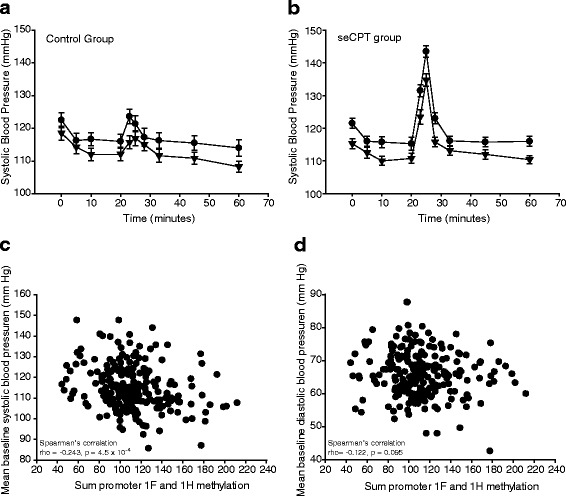
*NR3C1* promoter methylation effects on the systolic blood pressure response to the warm (control) or ice-cold water (seCPT) over the course of the experiment. Donors were split by median sum methylation levels. Systolic blood pressure in millimeter of mercury after the control (**a**) or cold water (**b**) was administered at 23 min and lasted 3 min. In both panels: *filled circles*, low methylation group; *filled triangles*, high methylation group. Data are the mean ± standard deviation. **c** Correlation between the mean baseline SBP and sum promoter 1F and 1H methylation levels. All participants are included, and each data point represents one participant. **d** Correlation between the mean baseline DBP and sum promoter 1F and 1H methylation levels. All participants are included, and each data point represents one participant. Baseline SBP and DBP mean of the three time-points immediately preceding the warm or cold water exposure

To evaluate the link between methylation of the two *GR* promoters studied and the stress response, a series of bivariate analyses, correlation tests and a focused principal component analysis were performed, identifying the factors that were subsequently used in a linear mixed effects model of the stress response (Table 4). SBP was identified as the variable to be explained, and *test group*, *methylation group*, *sex*, *arousal after the seCPT*, *arousal change*, *tension*, *discomfort*, *stress after the seCPT*, and *stress change* were retained as explanatory variables for further analysis. A maximum likelihood linear mixed effects model with an autoregressive matrix for the covariance structure of the residuals was constructed. Model residuals were normally distributed and centred on zero, suggesting a valid statistical model. This model confirmed the link between methylation levels and SBP, as well as having a significant effect on SBP evolution over time (Table 4). The interactions between seCPT group × time and methylation level × time were assessed but not significant (*p* > 0.05). A second mixed effects model was generated for DBP (Table 4). This model gave a similar distribution of the residuals and was equally valid. DBP was significantly associated with the methylation grouping and seCPT group (*p* = 0.019 and 0.031, respectively), although *time*, *arousal* and *stress* were not associated (*p* > 0.1). The effect of methylation group on SBP is illustrated in Fig. 5.

**Table 4:** Linear mixed effects models for systolic and diastolic blood pressure

	Value	SEM	DF	*t* value	*p* value
SBP					
Intercept	114.154	1.701	1864	67.115	0
High methylation group	**−**2.362	1.344	199	**−**1.757	0.0083
Time	**−**0.375	0.099	1864	**−**3.758	0.0002
Arousal	**−**0.052	0.037	199	**−**1.395	0.0761
Stress	0.028	0.035	199	0.805	0.3964
Test group (seCPT vs control)	2.313	1.652	199	1.400	0.2042
DBP					
Intercept	67.572	1.067	1864	63.350	0
High methylation group	**−**2.225	0.942	200	**−**2.361	0.0192
Time	**−**0.085	0.068	1864	**−**1.254	0.2101
Arousal	**−**0.03184	0.026	200	**−**1.203	0.2302
Stress	0.025	0.025	200	1.004	0.3164
Test group (seCPT vs control)	2.551	1.174	200	2.173	0.031

In the statistical model for both SBP and DBP, methylation data remained a valid predictor, despite the loss of power after dichotomisation. As a separate confirmation that the sum methylation of *GR* promoters 1F and 1H was significantly associated with peak SBP levels, methylation data was analysed as a continuous variable. Spearman’s correlations were performed, confirming this link (rho = −0.243, *p* = 0.00045; Fig. 5). However, DBP only had a trend towards associating with methylation (rho = −0.122, *p* = 0.095; Fig. 5).

### Association of haplotype and methylation levels

**Fig. 6:**
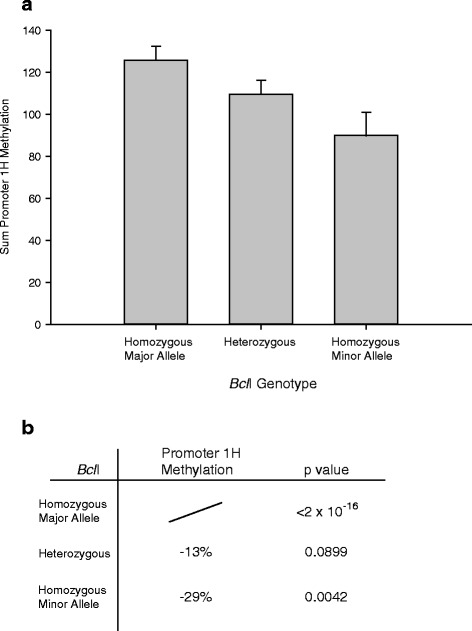
Statistical interpretation of the link between haplotype 2 (*Bcl*I alone) and promoter methylation. **a** Mean methylation level of donors separated by *Bcl*I genotype. **b** Summary linear association test results

Linear association tests revealed a link between the *Bcl*I minor allele and promoter 1H methylation (*p* = 0.00417; Fig. 6) although this was not significant for promoter 1F methylation. This link was confirmed using the chi-squared test on the median split methylation group, where the homozygous minor allele carriers were associated with promoter 1H methylation (*p* = 0.0423).

As the *Bcl*I genotype is part of haplotype 4 (*Bcl*I + *TthIII*I + 1H), the association between methylation and haplotype 4 was analysed. Haplotype 4 tended to associate with both promoter 1F and 1H methylation levels (linear association, *p* = 0.067 and 0.066) although the combined methylation grouping was not linked to the haplotype (*p* = 0.102, chi-squared test). Similarly, haplotype 5 (*TthIII*I + NR3C1-I + 9beta + ER22/23EK) showed an association trend to promoter 1F methylation levels (*p* = 0.064, linear association), but did not associate with promoter 1H methylation levels (*p* = 0.921, linear association).

As the *Bcl*I genotype has been previously reported to be in LD with variants in promoter 1H (rs10482614) [9]. Sanger sequencing of this promoter was performed. The minor alleles of rs10482614 and rs41423247 (*Bcl*I) were observable at frequencies of 29.3 and 36.2 %, respectively. As previously reported, rs10482614 was in LD with *Bcl*I (Cramer’s association coefficient, *V* = 0.324, *p* value = 3.032e−08, *r*^2^ = 0.16 and *d*’ = 0.77; Fig. 2). Although the presence of the minor allele of rs10482614 (G/A) removes a CpG dinucleotide, there was no significant link between the presence of the rs10482614 minor allele and methylation of the 1H promoter (*p* = 0.316).

## Discussion

The individual response to an external stressor is dependent on a panoply of factors. Here, we report the impact of *GR* promoter DNA methylation and sequence variants on the physiological response to stress. Increased *GR* 1F and 1H methylation levels were significantly associated with decreased baseline blood pressure. *GR* haplotype 2 (minor allele of *Bcl*I) carriers had a higher cortisol response to the seCPT. In addition, *GR* haplotype 2 carriers had higher heart rate and higher blood pressure independent of experimental group. Haplotype 3 carriers had a stronger heart rate decrease post stress. A major novel finding was that the *GR Bcl*I minor allele was associated with higher *GR* promoter 1H methylation.

The physiological response to stress involves the sympathetic nervous system (SNS) and the HPA axis. The cold pressor test (CPT), introduced by Hines and Brown, reliably increases blood pressure, a thermoregulatory reflex as well as a global activation of the sympathetic nervous system under standardised conditions [39]. Physiological responses, including vasoconstriction, increased skin conductance [40], and elevated blood pressure [41, 42] are induced. The addition of a social evaluative component in the seCPT adds a substantial HPA axis activation. However, rapid elevations in blood pressure trigger baroreflex mechanisms counteracting the heart rate increase. Consequently, blood pressure is considered the appropriate measure of cardiovascular reactivity in the seCPT [43], although we observed differences in both blood pressure and heart rate. The well-defined timing of the seCPT allowed us to successfully examine the baseline, immediate post-stress period and the return to baseline. At baseline, there were clear genetic and epigenetic effects on blood pressure. The seCPT induced a strong SNS and HPA axis response, and both systems were affected principally by genomic variants. The return to baseline was predominantly influenced by the genomic sequence.

Genomic variants had a significant effect on cardiovascular parameters. *GR* haplotype 2 (minor allele of *Bcl*I) carriers had higher baseline, peak and recovery period heart rate, and haplotype 3 carriers (minor allele of *TthIII*I, NR3C1-I and 9beta) had a stronger heart rate decrease post stress, both independent of the experimental group. Both of these haplotypes have previously been explored in detail, corresponding to haplotypes 4 and 2 from Cao-Lei et al. [9] and Kumsta et al. [38]. Haplotype 2 appears to play a central role in determining the cardiovascular stress response. However, *Bcl*I is an intronic polymorphism, 646 bp downstream of the common exon 2 that has generally been found to associate with increased GC sensitivity [11, 44], although the mechanisms are unknown. When the expanded haplotype 2 [9] is considered, around half of the carriers should also carry the minor alleles of the functional rs3806855 and rs3806854 in promoter 1B and rs10482614 in promoter 1H. In vitro, all three minor alleles reduced promoter 1H and 1B activity between 50 and 80 % [9]. Methylation of the entire 1B or 1H promoter had a similar effect, reducing promoter activity by up to 90 %. Logically, carrying haplotype 2 or having high promoter 1H methylation would have similar consequences including lower GR levels and increased cardiovascular stress reactivity and activity. Although only methylation level was associated with differential cardiovascular responses to seCPT, whereas *Bcl*I/haplotype 2 influenced heart rate independent of experimental group, there will be overlap in the mechanisms underlying their actions. Nevertheless, SNPs in a high LD with those investigated in this study might be regulators of methylation and physiological traits, especially since genetic variation that leads to methylation and expression variation at the same locus is not a rare phenomenon [45]. We hypothesise that the decreased promoter methylation observed in haplotype 2 carriers represents a counterbalance to the potential deleterious effects of the *Bcl*I genotype.

There is a well-established genetic component to variability in DNA methylation. Methylation quantitative trait loci (mQTL) are single genetic variants, often SNPs that correlate, or are associated with, DNA methylation levels. mQTLs operate over distances as large as 5 kb, occurring for approximately 2 % of the measured CpGs and 9.5 % of the expressed regions [45]. In contrast to Bromer et al. [36], we observed the *Bcl*I minor allele to correlate with high sum promoter 1F methylation levels. In our linear mixed effect model of the stress response, there was a significant interaction between methylation, genotype and cardiovascular activity. Sum methylation levels for promoter 1H and 1F + 1H were higher in women than men, and methylation levels were not normally distributed in either sex. Sex-specific DNA methylation profiles not unexpected as genome-wide levels are known to be higher in males [46, 47]. However, locus specific increases are not limited to males but have also been reported for women [48–51]. Similarly, increased age has been linked to both a reduction in global methylation levels, and dramatic genome-wide redistributions of 5-mC [52]. However, given the narrow age distribution of our participants, this was not observed. Although only methylation level was associated with differential cardiovascular responses to seCPT, whereas *Bcl*I influenced heart rate independent of experimental group, there might be a functional overlap between the two. Haplotype 2 and increased 1H methylation would both be expected to decrease promoter activity not only representing a specific *GR* mQTL, but also an expression methylation quantitative trait locus (emQTL) and even further a physiological expression methylation trait locus integrating the cardiovascular and stress responses with both genetic variants and methylation levels. Previous emQTL reports have all covered single CpG dinucleotides. There is currently contradicting data on the functional relevance of such limited methylation changes [53]. For the *GR*, we have previously shown that complete methylation throughout each proximal *GR* promoter efficiently inactivates them [9]. Similarly, methylation of a smaller (around 125 bp) fragment containing multiple CpGs also has functional effects, reducing promoter activity to ~25 % of the control, unmethylated sequence [5]. However, there is currently no evidence that methylation of a single CpG has functional consequences on *GR* expression. The importance of promoter-wide changes in DNA methylation is supported by recent clinical data from subjects suffering from posttraumatic stress disorder (PTSD). Whilst Lebonté et al. identified two CpGs in *GR* promoter 1F that associated with PTSD, Yehuda et al. nicely demonstrated that changes occurred promoter-wide [18, 54]. This is mirrored in both the rodent maternal care paradigm and the healthy human brain. Screening chromosome 18 that contains the rat GR, differential DNA methylation was observed in clusters across broad genomic regions [55]. At the individual CpG dinucleotide level strong distance-dependent correlations were found [15], further supporting our interpretation that DNA methylation changes occur in clusters and levels at individual CpGs are inter-dependent. These data lead us to suggest that our emQTL, unlike previous reports, is between haplotype 2 and a functionally relevant cluster of methylated CpGs in promoter 1H some 3 kbp upstream of the investigated region.

The generalizability and relevance of DNA methylation in peripheral blood samples to other tissues may appear questionable, as patterns are both locus and tissue specific. However, depending on the origin of the methylation patterns, it is probable that peripheral blood methylation levels are epigenetic proxies that mirror patterns in individual tissues of the cardiovascular system or the HPA axis. There are two plausible, non-exclusive mechanisms for this. Firstly, peripheral epigenetic variations may be the results of systemically acting circulating epigenetic modifiers such as cortisol [56]. Secondly, they may originate from a commonly programmed developmental precursor tissue. DNA methylation is established de novo during embryogenesis, when it is particularly susceptible to environmental influences. Epigenetic changes across primary germ layers occurring in this period will result in levels common to several differentiated tissues [57]. We have observed a strong correlation in methylation levels between ectoderm-derived tissues such as the anterior pituitary and the adrenal gland [3], as well as throughout the different neural tube derived tissues throughout the human brain [15] supporting the latter hypothesis. The corollary to this is that peripheral methylation levels may also be proxies for functional difference in GC sensitivity in other tissues from the same developmental origins.

The observation that haplotypes 2 and 3 have specific and different cardiovascular effects suggests that they act through different pathways. This concords with prior evidence that the renal pressure-natriuresis system and acute sympathetic activation mechanisms influence baseline cardiovascular traits and cardiovascular reactivity, respectively [58]. However, the role of GC and the GR in these mechanisms is unclear. In GC induced hypertension, pharmacological stimulation and receptor blocking data exclude direct GC/GR interactions [59, 60], suggesting indirect mechanisms such as oxidative stress or nitric oxide deficiency [61, 62]. Nevertheless, in vitro GC have significant effects on the NO system, including reducing endothelial and inducible NOS levels, reducing l-arginine and co-factor availability as well as inhibition of transmembrane l-arginine transport [63, 64]. Our data confirms this link between GC/GR and the cardiovascular response, albeit potentially via an indirect mechanism. There was a very clear link between SBP, to a lesser extent DBP, and methylation of promoter 1B and 1H. This was confirmed by the observation that *GR* haplotype 3 carriers had lower blood pressure after seCPT and a higher heart rate decrease. This implies that carriers of *GR* haplotype 3 may be protected against hypertension to some extent, even if there does not appear to be direct GC/GR involvement. Inversely, *GR* haplotype 2 had a significantly higher baseline heart rate. Indeed, the constituent *Bcl*I has been linked to hypertension [65]. Cortisol secretion (AUCg) was increased uniquely in carriers of the *Bcl*I containing haplotype 2. Our observations on the functional effects of haplotypes 2 and 3 can be generalised from our highly homogenous population to other populations, as these haplotypes have identical functional effects irrespective of ethnicity [66].

A weakness of our study is the limited number of SNPs that were investigated; however, the principal haplotypes previously established in the literature were readily identified. Similarly, the relatively small number of donors was counterbalanced by the high homogeneity of the young, infrequent-smoking, undergraduate student population reducing confounding socioeconomic factors.

## Conclusions

This is one of the first studies linking epigenetic modifications of the GR promoter, receptor genotype and physiological measures of the stress response. In the baseline period prior to the water task, there were clear genetic and epigenetic effects on blood pressure, particularly the *Bcl*I containing haplotype 2 and promoter 1F and 1H methylation. This was independent of the experimental group. The water task induced a strong cardiovascular and HPA axis response in the seCPT group and both systems were affected principally by the functional genetic variants. Methylation predicted lower SBP and DBP evolution over time in response to the water task. The return to baseline was predominantly influenced by the genomic sequence. The *Bcl*I polymorphism was associated with promoter 1H methylation levels. Promoter 1F methylation levels did not associate with any of the observed genetic variants, and as such are potentially influenced by the environment. Overall, we have shown that the induction and resolution of the stress response is controlled by an exquisite mix of genetic and epigenetic factors.

## Methods

### Participants

Participants were recruited from the University of Trier (Germany) via e-mail and poster advertisements as previously reported [37]. Briefly, 232 healthy non- and low-frequency smokers (<5 cigarettes per day) with a body mass index between 19 and 25 kg/m^2^ were recruited, and 218 (115 women and 103 men) completed the experimental protocol. Subjects with an increased objective or subjective sensitivity to cold and any indication of circulatory disturbances or cardiovascular problems were excluded. All participants completed the validated German version of The Beck Depression Inventory (BDI-II), and donors with scores above 19, consistent with moderate depression, were excluded [67–69]. As previously reported, caffeinated and alcoholic drinks, physical exercise and meals were not permitted in the 3 h immediately preceding the experimental visit [37]. All experiments were performed between 1:30 and 6 pm. In accordance to the declaration of Helsinki, the research was approved by the ethical committee of the medical association of Rhineland-Palatinate, and all participants gave their written informed consent.

### Socially evaluated cold pressor test

The socially evaluated cold pressor test (seCPT) was performed as previously reported [43, 70]. Briefly, participants assigned to the seCPT group were asked to completely immerse their hand in ice-cold (2–3 °C) water. Participants assigned to the control group were asked to completely immerse their hand in isothermic (35–37 °C) water. Participants in the seCPT group were under the social surveillance of an experimenter; their perceptions of social evaluation, uncertainty and lack of control were enhanced by warning them that the procedure may be painful, not communicating the duration of immersion during the test and informing them that their performance would be recorded for subsequent facial expression analysis. Participants assigned to the control group were not under social surveillance, and no video camera was present. All participants were asked to remove their hand from the water after 3 min. The sampling schedule is outlined in Additional file 1: Table S2. Immediately before and after cessation of the experiment, participants were asked to make a subjective rating of arousal, stress, anxiety, tension and activity on visual analogue scales ranging from 0 (“not at all”) to 100 (“very much”) in 10-point increments. Saliva samples were collected using absorbent cotton rolls (Salivette, Sarstedt, Nuembrecht, Germany). Samples were stored at −20 °C until analysis. Salivary cortisol was measured in duplicate using a time-resolved fluorescence immunoassay [71]. As previously reported, intra-assay and inter-assay coefficients of variance were 4.0–6.7 and 7.1–9.0 %, respectively [72]. Heart rate (HR) and blood pressure (SBP, DBP) were measured throughout the experiment using the Dinamap System (Critikon; Tampa, FL, USA) with the cuff placed on the right upper arm.

### Genetic analysis

#### DNA isolation

DNA was extracted from EDTA anti-coagulated blood using the salting out protocol of Miller et al. [73]. Genomic DNA concentration was measured on a NanoDrop 1000 spectrophotometer (NanoDrop Technologies, Rockland, DE, USA). DNA was stored at −20 °C prior to bisulfite modification and pyrosequencing or genotyping.

### Methylation analysis

Bisulfite modification and pyrosequencing were performed in duplicate as previously reported [15, 20, 74]. Briefly, 400-ng genomic DNA was bisulphite converted using the EpiTect-Bisulfite Kit (Qiagen) according to the manufacturer’s protocol. Promoters 1F and 1H were subsequently amplified by PCR and quantitatively pyrosequenced as previously reported [15, 20]. Pyrosequencing was performed using a PyroMark ID system, and methylation levels of each CpG dinucleotide was analysed using the Pyro Q-CpG software (version 1.0.9, Biotage). Positive controls were generated by incubation of genomic DNA with *Sss*I, and bisulphite conversion efficiency was calculated from the conversion rate of cytosine to thymidine when not immediately followed by a guanidine as previously described [15, 20].

### Genotyping and haplotype construction

The *GR* polymorphisms *TthIII*I (rs10052957), NR3C1-I (rs10482605), the promoter 1H SNP (rs10482614), ER22/23EK (rs6189 and rs6190), *Bcl*I (rs41423247) and 9beta (rs6198) were genotyped using a single nucleotide primer extension reaction, for which specific primers for each SNP were used in the SNPStart Master Mix kit from Beckman Coulter and where fragments were analysed with the CEQ8000 Genetic Analysis System (Beckman Coulter, Inc., Germany). Detailed information about primer sequences, PCR conditions and purification methods are available in supplementary information (Additional file 1: Table S2). Sanger sequencing of promoter 1H was performed as previously described [9]. All SNPs were tested for Hardy-Weinberg equilibrium. Linkage disequilibrium (LD) was assessed for all seven SNPs using Haploview 4.2 [75], and LD scores were expressed as D’. Individual haplotypes were reconstructed using PHASE, version 2.1 [76, 77] (http://stephenslab.uchicago.edu/software.html#phase), which uses an algorithm based on coalescence-based Bayesian haplotype inference for predicting haplotypes from genotype data, combining modelling strategy with computational strategies.

### Data reduction and statistical analysis

*GR* genotyping data was reduced by dichotymizing the SNPs with low minor allele frequencies combining the hetero- and homozygous carriers of the minor allele in one group. Cardiovascular data (HR, SBP, DBP) was reduced by extracting the mean increase of dependent variables from baseline to the peak after the water task and mean decrease from the peak to the recovery period. These are referred to as e.g. “SBP peak”, “SBP increase” and “SBP decrease”. Baseline was considered as average value of three measurements before water task. Recovery period was calculated using the three measurements after the water task. Cortisol data was reduced to the area under the curve with respect to ground (AUCg) and increase (AUCi; [78]). All variables were tested for normality graphically using kernel density plots and normal Q-Q plots and numerically using the Shapiro and Kolmogorov-Smirnov tests and the values of kurtosis and skewedness from the corresponding functions of the R package “moments”. Principal component (PCA) and internal consistency analysis (Cronbach’s alpha) were performed on all questionnaire derived data. Spherical representations of a correlation matrix and variance inflation factors (VIF) were used to identify correlations and co-linearity between covariates and explanatory variables. Variables with VIF >5 are considered co-linear and excluded from all subsequent models and analyses. Confounding factors were evaluated in a bivariate analysis for association with methylation status and test group using linear regression and Pearson’s or Spearman’s correlations or the chi-squared test, respectively. Any variable showing a significant association (*p* < 0.05) was included in a linear mixed effect model as a covariate. Linear mixed effects model selection was based on maximum likelihood, and an autoregressive matrix was chosen for the residuals covariance structure. All the analyses were performed using R, version 3.0.1 (The R foundation for Statistical Computing) except genotype and haplotype analyses for which general linear models (GLMs) were computed using SPSS 20.0 to assess the between-subjects effect genotype as well as the interaction time × genotype × groups for the cortisol level. Differences were considered to be significant when *p* < 0.05 after suitable post hoc correction in all statistical analyses. The Bonferroni correction was used for all bivariate analyses, and Tukey’s HSD was used for the repeated measures ANOVA (from R package Tukey HSD).

## Additional file

Additional file 1: Table S1–S3.
**Table S1.** Commonly reported haplotypes. **Table S2.** GR SNPs PCR primers and their reaction condition. **Table S3.** Association of GR gene SNPs and haplotypes with response of blood pressure, heart rate and cortisol response effect by SECPT.

## Abbreviations

AUC: area under the curve
DBP: diastolic blood pressure
GC: glucocorticoid
GR: glucocorticoid receptor
HPA: hypothalamus-pituitary-adrenal axis
HR: heart rate
LD: linkage disequilibrium
SBP: systolic blood pressure
seCPT: socially evaluated cold pressor test
SNP: single nucleotide polymorphism
UTR: untranslated region
