# Supplementary material for: The cardiovascular and hypothalamus-pituitary-adrenal axis response to stress is controlled by glucocorticoid receptor sequence variants and promoter methylation
Source: Clin Epigenetics. 2016 Jan 28;8:12. doi: 10.1186/s13148-016-0180-y (PMC4730588; doi:10.1186/s13148-016-0180-y)
Supplement: Additional file 1: Table S1–S3. — Table S1. Commonly reported haplotypes. Table S2. GR SNPs PCR primers and their reaction condition. Table S3. Association of GR gene SNPs and haplotypes with response of blood pressure, heart rate and cortisol response effect by SECPT. [file 13148_2016_180_MOESM1_ESM.pdf]

Supplementary Table 1 Commonly reported haplotypes

**Haplotype**	**Genotype Groups**	Previously Reported Haplotype Name / Number							
		a	b	c	d	e	f	g	h
Haplotype 1	Most Common Haplotype (MCH)	WT	1	1	1	1	1	1	1
Haplotype 2	*Bcl*I alone	*Bcl*I	4	4	2	1	4-1	2	2
Haplotype 3	*TthIII*I + NR3C1-I + 9beta			5		3	5	4	3
Haplotype 4	*ThIII*I + 1H + *Bcl*I					2	4-2	3	
Haplotype 5	*ThIII*I + NR3C1-I + 9beta + E22/23K	*ThIII*I + 9beta + E22/23K	2	2	4	4	2	5	6
Haplotype 6	*ThIII*I + *Bcl*I	*Thlll*I + *Bcl*I							4

[a]:Van Rossum, E.F.C., et al., Characterization of a promoter polymorphism in the glucocorticoid receptor gene and its relationship to three other polymorphisms. Clinical Endocrinology, 2004. 61(5): p. 573-581.

[b]: Kumsta, R., et al., Sex specific associations between common glucocorticoid receptor gene variants and hypothalamus-pituitary-adrenal axis responses to psychosocial stress. Biological Psychiatry, 2007. 62(8): p. 863-869.

[c]: Kumsta, R., et al., Characterization of a glucocorticoid receptor gene (GR, NR3C1) promoter polymorphism reveals functionality and extends a haplotype with putative clinical relevance. American Journal of Medical Genetics Part B: Neuropsychiatric Genetics, 2009. 150B(4): p. 476-482.

[d]: Otte, C., et al., Glucocorticoid receptor gene and depression in patients with coronary heart disease: The Heart and Soul Study—2009 Curt Richter Award Winner. Psychoneuroendocrinology, 2009. 34(10): p. 1574-1581.

[e]: Dekker, M.J.H.J., et al., Effect of glucocorticoid receptor gene polymorphisms in Guillain-Barré syndrome. Journal of the Peripheral Nervous System, 2009. 14(2): p. 75-83.

[f]: Cao-Lei, L., et al., Transcriptional control of the human glucocorticoid receptor: identification and analysis of alternative promoter regions. Human Genetics, 2011. 129(5): p. 533-543.

[g]: Spijker, A.T., et al., Glucocorticoid and mineralocorticoid receptor polymorphisms and clinical characteristics in bipolar disorder patients. Psychoneuroendocrinology, 2011. 36(10): p. 1460-1469.

[h]: Hardeveld, F., et al., Glucocorticoid and mineralocorticoid receptor polymorphisms and recurrence of major depressive disorder. Psychoneuroendocrinology, 2015. 55: p. 154-163.

Supplementary-Table 2: GR SNPs PCR primers and their reaction condition.

SNPs name	Sequence[a]	Tm[b] (°C)	Mg^{2+} (mM)	Primers (µM)
*TthIII*I	Fwd: 5'gtatttgttgggtgcctgct-3'<br>Rev: 5'actccagtgtgccagaaagg-3'	57.7	2.5	0.3
NR3C1-I	Fwd: 5'-aactcggtggccctcttaac-3'<br>Rev: 5'-aacctgttggtgacgcttg-3'	56.2	1.5	0.4
1H	Fwd: 5'-gccagaggtaagaagcgaggcggga-3'<br>Rev: 5'-gccggggcctccccggagcc-3'	70	0.7	0.1
E22E	Fwd: 5'-cagccgtgattgaaaagagg-3'<br>Rev: 5'-gccttttggaaaatcaacca-3'	56.3	2.0	0.3
R23K	Fwd: 5'-cagccgtgattgaaaagagg-3'<br>Rev: 5'-gccttttggaaaatcaacca-3'	56.3	2.0	0.3
*Bcl*I	Fwd: 5'-ttgctaaagcaatgcagtgaa-3'<br>Rev: 5'-tcaaacgaaagctgaaaattga-3'	57.7	2.5	0.4
9beta	Fwd: 5'tgactcctgtttaaaaataaaagttg-3'<br>Rev: 5'-cagattggacaatcggaactg-3'	57.7	2.5	0.4

[a]Fwd, forward or sense primer ; Rev, reverse or antisense primer. Primer with restriction enzyme recognition sites (underlined) used for amplification of the promoter fragments.

[b]Tm, annealing temperature in PCR.

SM_Table 3: Association of GR gene SNPs and Haplotypes with response of blood pressure, heart rate and cortiosol response effect by SECPT.

Genotypes and Haplotypes	*TthIII*I		NR3C1-1		1H		E22E		R23K		*Bcl*I (CC vs GG)		9beta		Haplotype1_CTGGGCA		Haplotype2_CTGGGGA		Haplotype3_TCGGGCG		Haplotype4_TTAGGGA		Haplotype5_TCGAACG		Haplotype6_TTGGGGA	
	maineffect[a]	interaction[b]	maineffect	interaction	maineffect	interaction	maineffect	interaction	maineffect	interaction	maineffect	interaction	maineffect	interaction	maineffect	interaction	maineffect	interaction	maineffect	interaction	maineffect	interaction	maineffect	interaction	maineffect	interaction
**Blood pressure**																												
SBP_basline												0,096						0,048							0,083	
SBP_recovery												0,075						0,067		0,019						
SBP_peak																										
SBP_ increase																						0,075				
SBP_ decrease																										
**Heart rate**																										
bpm_basline											0,048						0,022									
bpm_recovery											0,061						0,027									
bpm_peak																	0,023									
bpm_ increase												0,029														
bpm_ decrease																			0,016							
**Cortisol response**																										
AUCG																	0,053	0.034								
AUCI																										

[a] genotypes or haplotypes as maineffect factor

[b] the interaction is between genotypes or haplotypes and seCPT group.

P values are: unfilled squares p>0.1; hatched squares 0.1>p>0.05; filled squares p<0.05
